# Significance of Intra-abdominal Free Fluid Detected in Ultrasonography in the Clinical Assessment and Outcomes of Adult Patients Presenting to the Emergency Department Due to Abdominal Pain

**DOI:** 10.7759/cureus.5948

**Published:** 2019-10-21

**Authors:** Aylin Erkek, Yasemin Yılmaz Aydın, Handan Çiftçi, Hayri Ramadan, Kerim Temiz, Kuzey Aydınuraz, Figen Coskun

**Affiliations:** 1 Emergency Medicine, SBU Kocaeli Derince Training and Research Hospital, Kocaeli, TUR; 2 Emergency Medicine, Dışkapı Yıldırım Beyazıt Training and Research Hospital, Ankara, TUR; 3 Emergency Medicine, Kars Kafkas University Faculty of Medicine, Kars, TUR; 4 Emergency Medicine, Ankara Education and Research Hospital, Ankara, TUR; 5 Radiology, Samsun Vezirköprü State Hospital, Samsun, TUR; 6 General Surgery, Kırıkkale University Faculty of Medicine, Kırıkkale, TUR; 7 Emergency Medicine, Dokuz Eylül University Faculty of Medicine, İzmir, TUR

**Keywords:** acute abdominal pain, intraabdominal free fluid, ultrasonography

## Abstract

Objective

The aim of the study was to evaluate the diagnostic process and clinical course in adult patients who presented to the emergency department (ED) with acute abdominal pain (AAP) and were found to have intra-abdominal free fluid (FF) on ultrasonography (USG).

Methods

This prospective observational study was conducted in a training and research hospital adult emergency department between March 15, 2013, and April 15, 2013. The study included 252 patients aged above 18 years, who were admitted to the emergency room complaining of non-traumatic acute abdominal pain and provided consent for the study.

Results

The most common diagnoses were acute, nonspecific abdominal pain (37.3%), acute appendicitis (19%), and urinary tract pathology (15.9%). Intra-abdominal free fluid was detected with ultrasonography in 42.5% of patients. Patients with intra-abdominal free fluid were younger than the other patients. The emergency department length of stay was longer in patients with intra-abdominal free fluid (p=0.011). Of the 252 patients enrolled in the study, 32.9% were admitted to the hospital, 21.4% of whom underwent surgery and 11.5% received medical therapy. Most of the patients (64.5%) who were discharged home had no intra-abdominal free fluid in the ultrasonography (p<0.001).

Conclusion

The presence of intra-abdominal free fluid alone did not guide the clinical decision regarding the diagnostic evaluation of adult patients that presented to the emergency department complaining of non-traumatic acute abdominal pain.

## Introduction

Acute abdominal pain (AAP) is one of the most common causes of emergency department (ED) visits, accounting for approximately 5%-10% [1-2]. Medical history, physical examination, and laboratory and radiologic workup may not prove diagnostic at the initial assessment so follow-up is frequent, and only after this are 20% admitted to the hospital wards [2-3]. Although intra-abdominal free fluid (FF) can be the main finding of acute inflammation in non-traumatic AAP, it can also be detected in subjects without any physiological complaint [2-3]. The aim of the present study was to evaluate the diagnostic process and clinical course in adult patients who presented to the ED complaining of AAP and who were found to have intra-abdominal FF on ultrasonography.

## Materials and methods

This study was conducted in a training and research hospital in Turkey that has approximately 220,000 ED admissions per year. Patients with AAP that were admitted to the ED between March 15, 2013, and April 15, 2013, were evaluated. Patients aged above 18 years, with non-traumatic AAP and who provided consent for the study, enrolled. The exclusion criteria were pregnancy, known intra-abdominal malignancy, congestive heart failure, and undergoing an abdominal operation within the last month.

On-call physicians in the ED performed the clinical assessment. Age, gender, presence of a known disease, past intra-abdominal operation, drug use, menstrual cycle; characteristics, duration, location, and extension of abdominal pain; the presence of accompanying complaints, and conditions that aggravate or attenuate symptoms were documented. Tenderness on palpation, location of tenderness, guarding, and rebound tenderness were all recorded. A complete blood count was obtained in all patients. Basic metabolic profile and urine tests were obtained for the differential diagnosis.

Ultrasonography (USG) was performed by the same radiologist in all patients upon admission. The presence, amount, and location of intra-abdominal FF and pathological findings were recorded (Toshiba Xario USTS-770A, Tokyo, Japan; convex 3.5 MHz and flat 7.5 MHz probes).

ED length of stay, consultations, discharge and hospitalization status, medical or surgical treatments after hospitalization, and final diagnoses were recorded. Three days after discharge, the patients were called to check whether the pain persisted or they were admitted to or hospitalized in another health center.

The control group comprised 38 adult subjects among the hospital staff, without any abdominal pain, any other acute complaint, pregnancy, congestive heart failure, known intra-abdominal malignancy, or abdominal operation within the last month, and who provided consent for the study. They all underwent an ultrasonographical assessment by the same radiologist.

The statistical analysis was performed using the IBM SPSS for Windows version 21.0 software package (IBM Corp., Armonk, NY, US). Numeric variables were expressed as mean ± standard deviation and median [minimum - maximum] values. Categorical variables were expressed as number and percentage. The presence of a difference in categorical variables between the groups was investigated using the chi-square test and Fisher’s exact test. The normality of numeric variables was assessed using the Shapiro-Wilk test, and the homogeneity of variances was assessed using the Levene test. The differences in numeric variables between the two independent groups were tested using the t-test if parametric test assumptions were met. The Mann-Whitney U-test was used if parametric test assumptions were not met. The Kruskal-Wallis test was used to compare more than two independent groups. The level of significance was p<0.05.

The hospital ethics committee issued approval for the study protocol dated 06.03.2013, numbered 497, and with decision number 4110. The study was performed in accordance with the ethical standards laid down in the 1964 Declaration of Helsinki.

## Results

In 2013, 20,244 patients were admitted to the ED between March 15 and April 15; 1535 (7.6%) of whom were complaining of abdominal pain. The study included 275 patients with AAP who met the inclusion criteria and provided consent for participation in the study. Thirteen patients were lost to follow-up. Ten patients who were assessed ultrasonographically by another radiologist were excluded. The final study population consisted of 252 patients. The control group consisted of 38 healthy adults.

Characteristics of the study group

One hundred and fifty-nine (63.1%) were females. The mean age was 39.4±17.5 years (min-max: 18-87). Seventy-three percent of the patients did not have a known chronic disease while 18.7% had one, 6.7% had two, and 1.6% had at least three diseases. Seventy-one (28.2%) had undergone one to four prior abdominal surgeries.

Characteristics of the study group related to abdominal pain

The onset of pain was one to four hours in 51 (20.2%), and 25-48 hours in 120 patients (47.6%). Of the patients, 159 (63.1%) reported pain in one quadrant and 23 (9.1%) reported widespread pain in four quadrants. The right lower quadrant was the most common site of pain (123 patients, 48.2%) (Table 1).

**Table 1:** Pain characteristics of patients that were evaluated for acute abdominal pain

Feature	Value	
	n	%
Duration of pain (hour)		
1-4	51	20.2
5-24	21	8.3
25-48	120	47.6
49-120	60	23.8
Pain location upon admission		
Right lower quadrant	123	48.2
Right upper quadrant	53	21
Left lower quadrant	52	20.6
Umbilical	28	11.1
Epigastric	23	9.1
Widespread	23	9.1
Left upper quadrant	16	6.3
Suprapubic	13	5.2
Course of pain		
No change	124	49.2
Gradual Increase	103	40.9
Gradual Decrease	12	4.8
Colic	13	5.2
Presence of factors aggravating pain	150	59.5
Movement	119	47.2
Eating	38	15.1
Coughing	37	14.7
Lying on the back	23	9.1
Presence of alleviating factors	33	13.1
Lying on the back	24	9.5
Vomiting	10	4
Eating	3	1.2
Presence of complaints accompanying pain	153	60.7
Nausea	95	37.7
Vomiting	62	24.6
Lack of appetite	39	15.5
Dysuria	29	11.5
Pollakiuria	18	7.1
Constipation	16	6.3
Diarrhea	15	6
Fever	9	3.6

Abdominal physical examination findings of the study group

The abdomen was tender to palpation in a single quadrant in 152 patients (61.5%), and there was widespread tenderness in four quadrants in 17 patients (6.7%) (Table 2).

**Table 2:** Physical examination findings on admission in patients that were evaluated for acute abdominal pain

Feature	Value	
	n	%
Appearance of the abdomen		
Presence of scar	66	26.2
Presence of distension	2	0.8
Bowel sounds		
Normal	245	97.2
Decreased	5	2
Increased	2	0.8
Tenderness to palpation		
Single area	155	61.5
2 areas	75	29.8
3 areas	5	2
Widespread	17	6.7
Location of tenderness tenderness		
Right lower quadrant	143	56.7
Left lower quadrant	54	21.4
Right upper quadrant	47	18.7
Umbilical area	32	12.7
Mc-Burney’s point	22	8.7
Suprapubic area	17	6.7
Widespread in all quadrants	17	6.7
Murphy’s point	16	6.3
Left upper quadrant	14	5.6
Epigastric area	14	5.6
Costovertebral angle tenderness	15	6
Presence of Guarding	52	20.6
Presence of Rebound Tenderness	97	38.5

Laboratory and imaging findings of the study group

A hemogram was obtained from all patients at the time of admission, from 55 patients (21.8%) at four hours of the follow-up period, and from three patients (0.12%) at eight hours of the follow-up period. All patients underwent an abdominal USG. The characteristics and diagnoses are given in Table 3 and Table 4.

**Table 3:** Laboratory findings of patients that were evaluated for acute abdominal pain

Feature	Value	
	Mean±SD	Min.-Max.
White blood cell count /mm3		
On admission	11233±4236	4460-31000
At 4 hours (n=55)	11500±4025	5100-22800
At 8 hours (n=3)	11066±2610	8600-13800
Haematocrit		
On admission	41.7±5.2	15.9-57.3
At 4 hours (n=55)	39.5±5.2	28.9-54
At 8 hours (n=3)	41.2±1.3	40.4-42.8
Presence of leukocytosis (>11000/ mm3)	n	%
On admission	105	41.7
At 4 hours	27	10.7
At 8 hours	1	0.4
Elevated alanine aminotransferase level (>100 IU)		
On admission	6	2.4
At 4 hours	2	0.8
Elevated aspartate aminotransferase level (>100 IU)		
On admission	8	3.2
At 4 hours	2	0.8
Elevated amylase Level (>200 IU)		
On admission	10	4
At 4 hours	2	0.8
Urine analysis	170	67.5
Erythrocyturia	50	19.8
Leukocyturia	70	27.8
Nitrite positivity	1	0.4
Ketone positivity	7	2.8

**Table 4:** Characteristics of free fluid in the ultrasonography assessment of patients in the study group USG: Ultrasonography

Feature	Value	
	n	%
Free fluid in the USG (+)	107	42.5
Quantity of free fluid in the USG		
Mild	33	30.8
Minimal (<2 cm)	62	57.9
Moderate (2-5 cm)	11	10.3
Severe (>5 cm)	1	0.9
Localization of free fluid in the USG		
Between bowel loops	68	63.6
Douglas pouch	10	9.3
Pelvic region	6	5.6
Pericholecystic area	3	2.8
Pararenal area	2	1.9
Paraovarian area	2	1.9
Perivesical area	1	0.9
Peripancreatic area	1	0.9
Between bowel loops + Douglas pouch	9	8.4
Between bowel loops + Pericholecystic area	2	1.9
Between bowel loops + Gastrohepatic	1	0.9
Between bowel loops + Paraovarian area	1	0.9
Between bowel loops + Douglas pouch + Paraovarian area	1	0.9

Findings related to follow-up and treatment in the study group

Nonspecific abdominal pain was most common (94 patients, 37.3%), followed by acute appendicitis (48 patients, 19%), urinary tract pathology (40 patients, 15.9%), gynecologic disorders (22 patients, 8.7%), gall bladder diseases (18 patients, 7.1%), bowel pathologies requiring surgery (9 patients, 3.6%), acute pancreatitis (6 patients, 2.4%), ischaemic pathologies (2 patients, 0.8%), abdominal mass and abscess (2 patients, 0.8%), diverticulitis (2 patients, 0.8%), and other abdominal pathologies (9 patients, 3.6%). The definitive diagnosis after surgery was acute appendicitis in 43 patients (17.1%). Other data are given in Table 5.

**Table 5:** Emergency room follow-up and assessment data of patients in the study group

Feature	Value	
	n	%
Emergency Room Length of Stay		
1-2 hours	26	10.3
3-4 hours	156	61.9
5-8 hours	60	23.8
>8 hours	10	4
Consultation (+)	165	65.5
Requested Consultation		
General Surgery	139	55.2
Gynecology	54	21.4
Other Branches	8	3.2
Outcomes in the emergency room		
Discharged from the emergency room	162	64.3
Hospitalized	83	32.9
Left with their own wish	7	2.8
Treatment of hospitalized patients		
Surgery	54	65.1
Medical therapy	29	34.9

On Day 3 after discharge, all 169 patients were called to obtain information about their clinical condition. Forty-eight patients (19%) were admitted to another healthcare facility due to abdominal pain. Twenty-six (10.3%) received medical therapy, whereas two patients (0.8%) underwent surgery (one cholecystitis and one appendicitis). Fourteen patients (5.6%) continued outpatient control visits and two patients (0.8%) were hospitalized in another center for medical therapy. Of these patients, 4.4% (n=11) had not been diagnosed yet.

Comparison and analysis of the data of the study group

Patients with intra-abdominal FF in USG were younger while the FF incidence was lower in postmenopausal women (p<0.000, p<0.000). In female patients, FF was located between the bowel loops and pelvis (p<0.000).

The majority of patients with findings suggestive of a non-surgical or infectious condition did not have FF (p=0.03). FF was more common in patients with an aggravating factor and without an alleviating factor (p=0.031, p=0.038). FF accompanied by a pathological diagnosis was significant for older patients (p=0.002). The presence and location of FF, as well as abdominal tenderness, were more significant in patients with right upper quadrant pain (p<0.00, p=0.006, p=0.015). FF was accompanied by guarding (p=0.019) and rebound tenderness (p=0.041). FF was associated with both air-fluid levels on plain abdominal X-ray and the presence of a pathological diagnosis (p=0.035, p=0.02).

There was no FF in the majority of patients with nonspecific abdominal pain and patients who were discharged home while there was FF in the majority of patients diagnosed with a gynecologic disorder (p<0.01, p<0.00, p<0.001). FF alone was more common in patients who received medical therapy (p=0.02).

The majority of patients who stayed one to two hours in the ED had no FF (p=0.011). Consultation requests and hospitalization were more frequent in patients with FF and ultrasonographical pathological diagnosis than the detection of FF only (p=0.008, p<0,001).

Ultrasonographical pathology appeared to be the major factor leading to the decision to perform surgery (p=0.033). Twenty-two patients with FF and a pathological diagnosis (55%), six patients with FF but without a pathological diagnosis (66.7%), and 21 patients without FF but with a pathological diagnosis (84%) underwent surgery. Patients with ultrasonographical detection of both FF and pathologic diagnosis had significant re-admission rates (p<0.001) (Table 6).

**Table 6:** Presence or absence of free fluid in treatment decisions

Feature	Presence of Free fluid					
	positive			negative		
Consultation request	n	%		n	%	p
Requested	96	58.2		69	41.8	0.00٭
Not requested	11	12.6		76	87.4	
Hospitalization decision						
Discharged	56	34.6		106	65.4	0.00٭
Hospitalization	49	59		34	41	
Inpatient treatment						
Operated	27	50	27		50	0.02*
Medical therapy	22	75.9	7		24.1	
Admission to another healthcare facility						
Yes	17	35.4	31		64.6	0.992
No	41	33.9	80		66.1	

Characteristics of the control group

Of the 38 patients in the control group, 21 (55.3%) were females. The mean age was 29.1±10.28 years (min-max=18-56). There was no FF in 36 subjects (94.7%) in the control group, a finding different from the patient group (p<0.000). Of the two patients with FF, only one had ultrasonographically detected pathology, which was an ovarian cyst.

## Discussion

AAP is one of the most common causes of ED visits. Intra-abdominal FF is one of the findings of acute inflammation in non-traumatic AAP but it can also be detected in patients without any complaints [1-2,4]. The demographic properties of the study population were consistent with studies by Hastings et al. and Cooper et al., reporting female gender predominance and similar figures for incidence, mean age of AAP admissions, as well as pain distribution [5-6]. Guarding was present in 20% and rebound tenderness in 38% of patients, with 57.4% guarding and 92.6% rebound tenderness present in patients who underwent surgery in accordance with the literature [7-8]. The majority of patients requiring emergency surgery or hospitalization had leucocytosis, similar to the study by Çalışkan et al [8]. In a meta-analysis by Andersson et al., the positive likelihood ratio was 2.47 for the diagnosis of acute appendicitis [9]. Follow-up was one to four hours in 72.2% of the patients, similar to 65.9% in a study by Aygencel et al. In the present study, 32.9% of patients were hospitalized, 21.4% of which required surgery consistent with the literature [3]. Different rates of hospitalization are reported in studies by Hastings et al. and Lindelius et al. [4,10].

USG is an important imaging method for AAP due to its low cost, easy accessibility, lack of radiation exposure, and contrast agent with the rate of accurate diagnosis increasing from 70% to 83% when USG is performed after clinical assessment [11].

In the present study, USG showed normal findings in 59.5% of the patients, which is higher than the incidence of normal ultrasonographical findings in other studies [12]. Acute appendicitis (14.3%), gynecologic problems (8.7%), and gall bladder problems (7.1%) were the most frequent diagnoses in our study.

Intra-abdominal FF observed on imaging could also be physiologic. FF frequency was 30%-40% in healthy female volunteers of reproductive age [13-14]. Brown and Dubbins reported intra-abdominal FF in 40% of healthy voluntary males [15]. In the present study, USG showed FF in two out of 38 subjects in the control group (5.3%).

Patients with FF were significantly younger in our study while patients with pathological findings and FF were older. Rathaus et al. evaluated intra-abdominal FF in 266 patients with acute and/or chronic abdominal pain versus 396 asymptomatic pediatric patients and reported a significantly higher rate of FF in children with abdominal pain (28.6%) as compared to asymptomatic children (7%). Gender did not affect the presence of FF in the present study, similar to results by Rathaus et al. [16]. Patients that had FF in the Douglas and pelvic areas were also significantly younger while 85.4% of postmenopausal women did not have FF. The presence of FF was not affected by gynecologic factors in our study despite studies reporting an increase in physiologic FF in asymptomatic adult females in the ovulatory period [17].

It can be argued that the absence of guarding and rebound tenderness may be suggestive of the absence of FF, as USG failed to show FF in the majority of patients showing no guarding and rebound tenderness.

FF was mostly detected within the bowel loops (63.6%), Douglas pouch (9.3%), and pelvic area (5.6%). The predominance of fluid accumulation within the bowel loops is consistent with the results of a study by Jequier et al. in which FF was mostly located within the bowel loops (68%) again [18].

In our study, 65% of patients without any pathological finding did not have FF. The majority of patients with normal USG or findings suggestive of a non-surgical or infectious condition did not have FF while FF was common in patients with a gynecologic or urologic condition. Similarly, in the study by Matz et al., the final diagnosis was of gynecologic or urologic origin in 36% of patients who had intra-abdominal FF alone [19].

In our study, there was a difference in consultation requests depending on the presence of FF and ultrasonographical pathological findings. The presence of FF did not indicate a requirement for consultation; however, 87.4% of patients for which consultation was not requested did not have FF. On the other hand, a consultation was mostly requested in the presence of FF accompanied by ultrasonographically diagnosed pathology (98.2%). In the present study, the absence of FF affected the ED length of stay. Seventy-six point nine percent (76.9%) of patients with one to two hours of ED had no intra-abdominal FF.

USG did not show FF in 64.5% of discharged patients. The presence of FF did not favor surgical therapy versus medical therapy and 75.9% of patients with FF alone received medical therapy after hospitalization.

The hospitalization rate was 74.1% for patients with both FF and pathological conditions while it was 17.6% for patients with FF alone. Matz et al. found a discharge rate of 48.8% for patients with FF but without a confirmed surgical pathology [19]. Also, the presence of FF alone did not affect re-admission to another health care facility (p=0.99).

In our study, FF was present in 42.5% of 252 patients who presented to the ED due to non-traumatic acute abdominal pain. The clinical significance of intra-abdominal FF was investigated in 407 pediatric patients with AAP in the study by Matz et al. where FF could be visualized in 33% of the patients. They investigated the importance of intra-abdominal FF in leading to the decision of surgery and found that intra-abdominal FF alone did not influence the decision of surgery in the absence of a pathological finding on imaging studies [19]. In their study group, 40.3% of patients with intra-abdominal FF plus confirmed surgical pathology underwent surgery while only 6.3% of children with intra-abdominal FF alone underwent surgery [19].

Limitations

The present study may have limitations due to the fact that only patients admitted to the ED of one hospital and patients who were ultrasonographically assessed by a single radiologist were included in the study.

## Conclusions

The present study is an original study that evaluated FF using USG in adult patients with non-traumatic acute abdominal pain. Ultrasonographical FF did not necessarily indicate an intra-abdominal pathology and did not favor surgical therapy as compared to medical therapy. Of the patients with FF but without a detectable pathology, 82% were discharged from the ED. The presence of intra-abdominal FF alone was not found to guide the assessment of adult patients with non-traumatic abdominal pain.
